# Clozapine and desmethylclozapine: correlation with neutrophils and leucocytes counting in Mexican patients with schizophrenia

**DOI:** 10.1186/s12888-019-2286-1

**Published:** 2019-10-09

**Authors:** Mayela Vaquero-Baez, Araceli Díaz-Ruíz, Luis Tristán-López, Carlos Aviña-Cervantes, Carlos Torner, Jesús Ramírez-Bermúdez, Sergio Montes, Camilo Ríos

**Affiliations:** 1 Doctorado en Ciencias Biologicas y de la Salud, Universidad Autonoma Metropolitana-Xochimilco, Ciudad de México, México; 2 Departamento de Neuroquímica, Instituto Nacional de Neurología “Dr Manuel Velasco Suarez”, Ave. Insurgentes Sur No. 3877, 14269 Ciudad de México, D.F México; 3 División de Psiquiatría, Instituto Nacional de Neurología, “Dr Manuel Velasco Suarez”, Ciudad de México, México; 4 Laboratorio de Neurofarmacología Molecular, Departamento de Sistemas Biológicos, Universidad Autónoma Metropolitana-Xochimilco, Ciudad de México, México

**Keywords:** Clozapine, N-desmethyl clozapine, Neutrophils, Treatment-resistant schizophrenia

## Abstract

**Purpose:**

The aim of present study is to measure plasma clozapine (CLZ) and N-desmethyl clozapine (DMC) as biomarkers to correlate drug concentrations with the appearance of preclinical adverse hematic effects.

**Methods:**

A high-performance liquid chromatographic method, using a diode-array (ultraviolet) detector, was validated to obtain reliable concentrations of CLZ and DMC, its main metabolite, in plasma of 41 schizophrenic patients taking CLZ. Blood neutrophils and leucocytes counting were concurrently assessed as a proxy to subclinical adverse reactions.

**Results:**

The analytical method employed was linear, reproducible, and stable to measure concentrations of CLZ between 30 and 1000 ng/mL, while 12.5–560 ng/mL of the metabolite. The method allowed us to correlate CLZ plasma concentrations, the time taking CLZ and CLZ dose as determinants of neutrophils’ counting with a R^2^ = 0.447, using a multiple regression analysis model. Likewise, the correlation of leucocyte counting vs CLZ plasma levels and CLZ time, showed a R^2^ = 0.461. DMC correlated significantly with both neutrophils and leucocytes counting, but was excluded from the regression when CLZ concentration was included in the model. Finally, no other hematological adverse reactions were recorded. One patient presented a cardiovascular complication. The negative correlation between clozapine and neutrophil count observed in patients, suggest that CLZ itself, but not DMC, could be related to hematologic side-effects.

**Conclusion:**

The findings of this study, demonstrate for the first time, that plasma levels of CLZ and time taking the drug are independent determinants of blood neutrophils and leucocytes, so the monitoring of plasma CLZ may be useful in the clinic practice to determine safe dosing of the drug.

## Background

Schizophrenia is a chronic and debilitating disease, affecting approximately 0.5% of the world population [1]. It is a treatable condition, with many therapeutic alternatives, nevertheless, 20–30% fail to respond to treatment, and a similar fraction of treatment adherent patients relapse despite sustained maintenance therapy [2]. Treatment-resistant schizophrenia (TRS) is defined in clinical guidelines as a situation in which a significant improvement in psychopathology has not been demonstrated despite two or more different antipsychotic treatment trials, each with adequate dose and duration [3].

CLZ, a dibenzodiazepine developed in 1960’s, is considered an atypical antipsychotic, approved for the treatment of TRS [4]. It has been demonstrated that clozapine is more effective than any other first- generation agents (FGA) or second-generation antipsychotics (SGA) in the treatment of resistant schizophrenia. Even, it has been estimated that almost two thirds of those patients who do not respond adequately to treatment with FGAs or other SGAs, may respond adequately to treatment with clozapine [5].

The CLZ causes lower extrapyramidal symptoms from the side effects caused by neuroleptic drugs, mainly tardive dyskinesia [6]. However, the use of CLZ has been associated to agranulocytosis reported in 0.38% (995 cases) of treated patients, 12 deaths attributed to complications of agranulocytosis [7]. Some studies have shown that the stable active metabolites of CLZ, such as N-desmethyl clozapine (DMC), may related to the neutrophil counting in patients. The mechanism of neutrophil decline in patients treated with clozapine is still poorly understood. It has been suggested that clozapine metabolism involves an unstable reactive metabolite, nitrenium ion that is thought to bind covalently to macromolecules such as proteins or causing overproduction of reactive oxygen species. Under in vitro conditions, DMC has been reported to be more toxic than clozapine, but the concentrations of DMC used in that study were far above those obtained by clozapine metabolism under therapeutic dosing [8]. Reactive oxygen species and nitrenium ion neutralization both involve the use of reduced glutathione; noteworthy, in patients with refractory schizophrenia, the circulating glutathione levels are decreased as compared to control subjects [9].

The pharmacological treatment efficacy of CLZ in schizophrenic patients must be evaluated by the psychiatrist through clinical evaluation. However, the adherence to the clinical prescription is essential to be successful in the treatment. In this regard, 54% of the patients with schizophrenia or bipolar disorder reported intentional non-adherence [10], therefore, in those patients, the vigilance made by the pharmacist, to assess the compliance to medical indications in order to increase the probability of clinical success.

CLZ is a good option for pharmacological refractory schizophrenic patients and its popularity is increased [11]. It has been considered the gold standard treatment for both positive and negative refractory symptoms in schizophrenia [12]. However, the incidence of agranulocytosis and other hematic adverse effects, encourage the health professionals to put special attention to perform pharmacovigilance and to study the mechanism underlying such phenomenon.

The aim of the present study was to study the relationship between CLZ and the its main pharmacologically active metabolite, DMC plasma levels with neutrophils and leucocytes counting in schizophrenic patients taking CLZ, in order to explore the possibility that those variables are related, before a severe toxicological condition is displayed by patients.

## Methods

### Patients and study design

Forty-one patients, diagnosed with schizophrenia at the Neuropsychiatry Clinic of the National Institute of Neurology and Neurosurgery in Mexico City, were invited to participate in the study, which was conducted according to the Good Clinical Practices, the Declaration of Helsinki (October 1996 version) and with the approval of the local Institutional Bioethics Committee with registry number 92/07. Written informed consent to participate was obtained from all subjects before enrollment in the study, from a relative or guardian for each patient and two witnesses, according to Mexican Code of Health Regulations. The schizophrenia diagnosis was performed according to the Structured Clinical Interview (SCID) for DSM-V. The inclusion criteria were: either men or women, with diagnosis of refractory schizophrenia treated chronically with CLZ, older than 18. The exclusion criteria were: patients with another psychiatry illness, patients with a history of substance abuse in the last 3 months except tobacco, occasional consumption of alcohol or marijuana, and patients with severe anemia or with pregnancy.

The psychiatrist evaluated the efficacy of the treatment by clinical parameters (PANSS scale). The pharmacist offered treatment and illness education to patients and their families and looked for adverse reactions, evaluated the psychiatrist’s prescription, counted the patient’s medication and evaluated the safety of pharmacological treatment. CLZ treatment started with 25 mg/day dose and escalated up to 900 mg/day, according to the specialist directions. Blood samples were collected by venous puncture and centrifuged to obtain plasma samples that were stored at − 20 °C until analyzed. Clinical variables were also recorded for each patient, such as: concomitant drug treatment, and the time patients were under CLZ medication (Time taking CLZ). Even when the study is cross-sectional, the inclusion of a time variable allowed us to perform an evaluation of the impact of time on blood cells ‘counting. The time taking CLZ is also an indirect assessment of the cumulative dose taken by each patient.

### Chromatographic analysis

We used a chromatographic system (Agilent technologies, model 1100), equipped with a diode array detector. The wavelength was set at 245 nm and 100 μl volume injection. An analytical column Brava Cyano, 5 μm, 250 mm × 4.6 mm (Grace, Alltech) was used. The mobile phase consisted of acetonitrile/ammonium acetate buffer 1 M (36:64 v/v), pH 5.7 and flow adjusted to 1 ml/min.

We use the protriptyline as internal standard, 250 ng/μl, clozapine and N-desmethylclozapine. Sample preparation was performed as reported by Volpicelli et al. [13] with minor modifications. The analytical method was previously validated for linearity, precision, and accuracy. We analyzed recovery, selectivity and the stability for the samples after 90 days [14].

### Blood neutrophils and leukocytes counting

The neutrophils and leukocytes counting were performed at the clinical laboratory of the National Institute of Neurology and Neurosurgery. All blood samples were assessed using a XN-1000 Sysmex hematology analyzer, according to the manufacturer’s protocol.

### Statistical analyses

Data were analyzed by using statistical software SPSS (IBM. Corp. Released 2015. IBM SPSS Statistics for Windows, Version 23.0. Armonk, NY. USA: IBM Corp). Exploratory analysis was applied to data in order to check for normality distribution. Bivariate analyses were then applied to select variables association, using bivariate Spearman’s and Pearson’s correlations.

For the construction of multiple regression models, bivariate analyses in the form of simple lineal regression were first carried out to select variables. For those analyses, variables were selected when *p* < 0.05 in one-tailed tests. Multivariate modeling was done through least-squared multiple regression, considering neutrophils or leukocyte counts as dependent variables, and plasma concentrations of CLZ, DMC, age, time taking CLZ, and CLZ dose, as independent ones.

The assumption of normality of the data, after applying multiple regression models, was verified by residual analysis plots.

## Results

### Chromatographic analysis

**Fig. 1:**
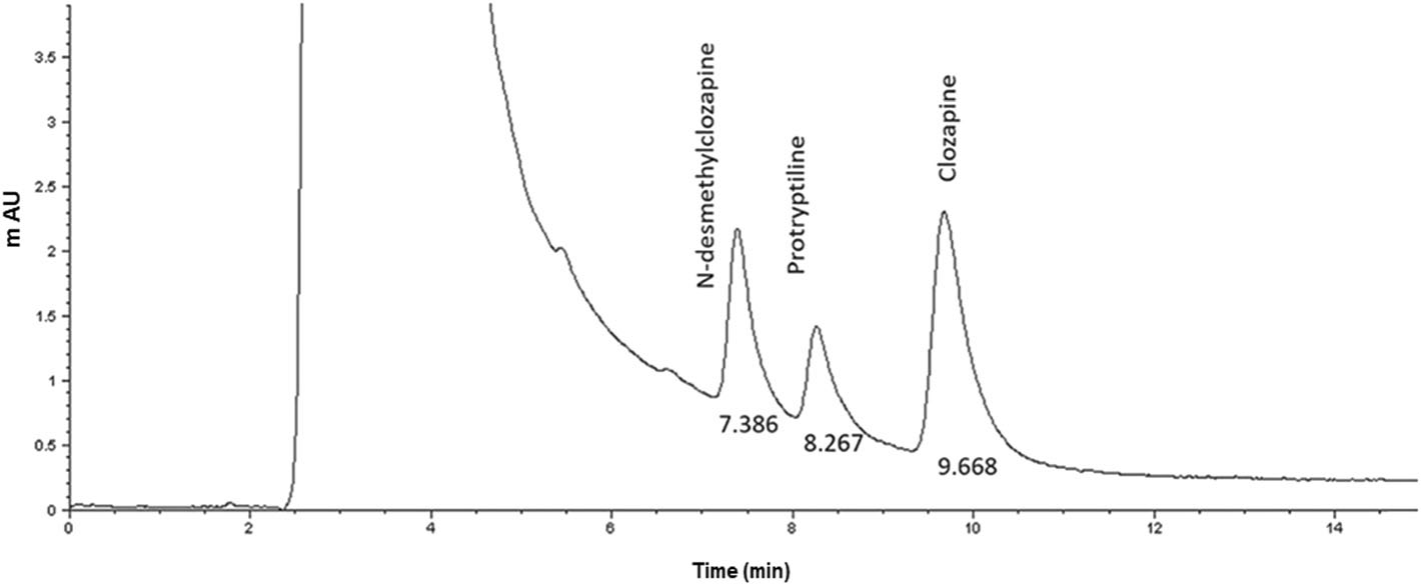
Representative chromatogram showing the separation of Clozapine, Desmethyl Clozapine and the internal standard, Protryptyline

The HPLC-UV method was linear, exact, intra inter-day precision and stable to measure concentrations of CLZ between 30 and 1000 ng/mL, while 12.5–560 ng/mL of the metabolite. It was a selective method and also allowed the adequate resolution of internal standard, from CLZ and DMC, see Fig. 1. The intermediate mean recovery points ranged − 3.36 to 0.06% for CLZ and − 0.39 to − 1.02% for DMC.

The absolute deviation was within a range of − 5.61 to − 0.39% of absolute deviation for CLZ and − 5.66 to 4.59% of absolute deviation for DMC.

From the stability study, plasma samples and stock solutions of CLZ and DMC were stable for 90 days at − 70 °C, the absolute deviation has a range of − 4.18 to − 0.33% and − 5.66 to 4.59%, respectively. The stability study demonstrated that plasma samples can be maintained frozen, for up to 1 month without change in their CLZ or DMC concentrations.

### Clinical features of selected patients

Forty-one patients were included in this study and the descriptive statistics are shown in the Table 1. Fifty-percent of the subjects showed concomitant illnesses, the more frequent of those was depression. Twelve percent of patients underwent a non-pharmacological treatment, a regional tea (tila tea), and massages in the head with relaxing oils. The concomitant medications taken by the patients are described in Table 2. None of them modified the effect of CLZ on cells’ counting.

**Table 1:** Descriptive statistics of 41 patients treated with CLZ

Parameters	N	Mean ± SE	Median (range)
Age (years)			
Male	22 (53.7%)	34.68 ± 1.58	36 (23–50)
Female	19 (46.3%)	31.58 ± 1.29	35 (26–43)
CLZ time (months)			
Male		10.05 ± 1.74	10 (1–32)
Female		6.63 ± 1.15	6 (1–24)
CLZ dose (mg/day)			
Male		222.73 ± 42.95	150 (100–900)
Female		105.26 ± 8.46	100 (50–200)
CLZ levels (ng/mL)			
Male		290.11 ± 51.56	203.25 (44–846)
Female		336.36 ± 29.16	321.77 (91–572)
DMC levels (ng/mL)			
Male		134.98 ± 12.78	108.93 (28–308)
Female		127.17 ± 16.91	133.78 (47–245)
		Number of subjects (percentage)	
Tobacco smokers		12.2% (*n* = 5)	
Marijuana smokers		4.9% (*n* = 2)	
Drink alcohol (alcoholism)		7.3% (*n* = 3)	
Drink coffee		14.6% (*n* = 6)

**Table 2:** Pharmacological concomitant medication

Drug	Percentage of patients
Clonazepam	16 (30%)
Fluoxetine	12 (22%)
Paroxetine	5 (9%)
Lithium	4 (7%)
Mirtazapine	3 (6%)
Metformin	2 (4%)
Sulpiride	2 (4%)
Valproate magnesium	2 (4%)
Venlafaxine	2 (4%)
Duloxetine	1 (2%)
Escitalopram	1 (2%)
Imipramine	1 (2%)
Losartan	1 (2%)
Omeprazole	1 (2%)
Pregabalin	1 (2%)

Three adverse reactions were observed: only one was severe, detected as an increased QT interval for which the psychiatrist decreased the dose of CLZ employed, returning the QT interval to normality after 1 week. The pharmaceutical accessories helped the patients to comply adherence to treatment, in this study the partial adherence was 9.5%, lower than that reported by studies in the literature [10, 15]. Fifty-four percent of patients were back to work after 10 weeks on average clozapine treatment.

### Bivariate determinants of neutrophils and leucocytes counting

**Fig. 2:**
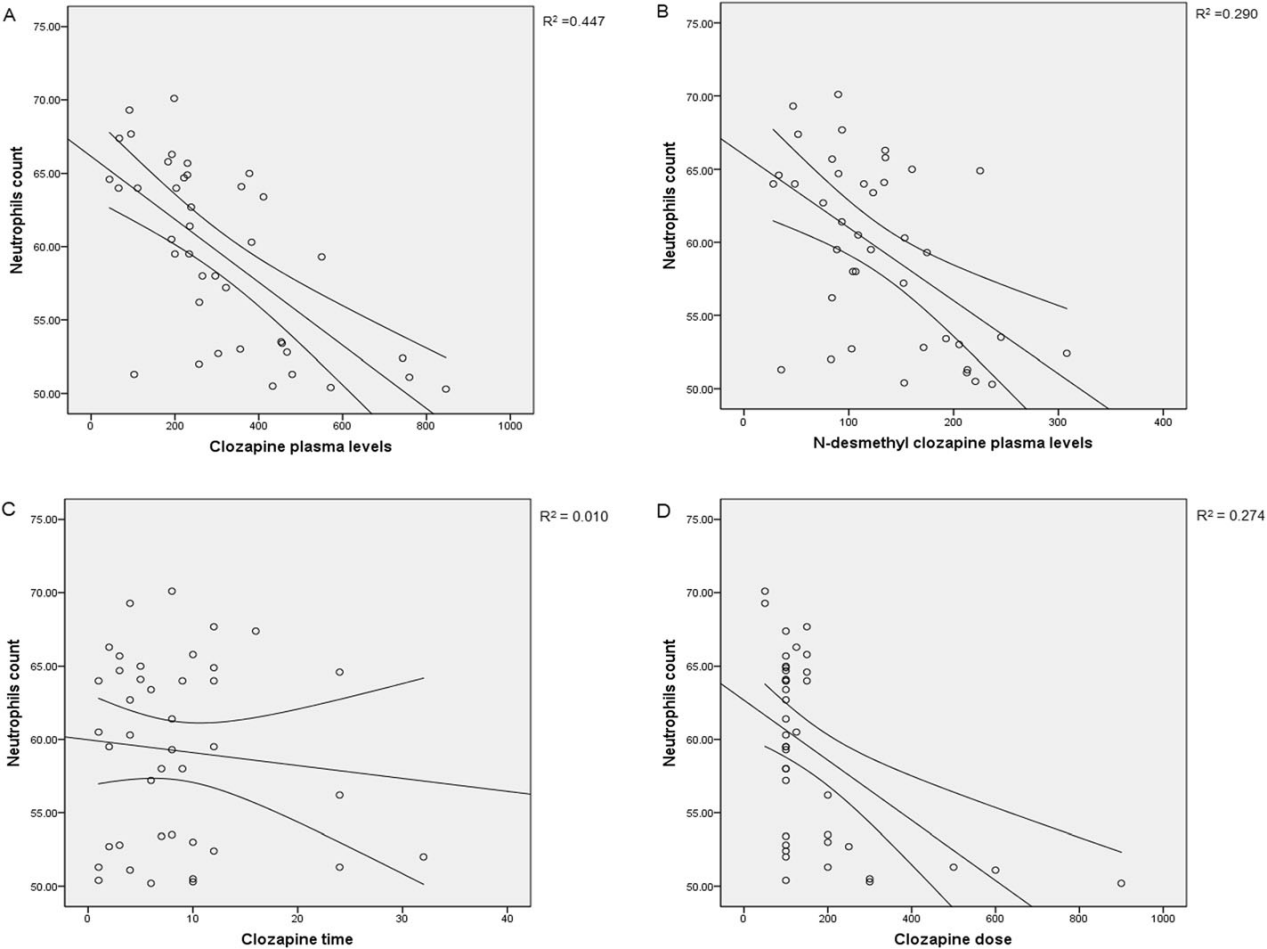
Scatter plot showing the main relationships between neutrophils counting and CLZ or DMC. Spearman’s and Pearson’s correlation and linear regression analysis were applied

**Fig. 3:**
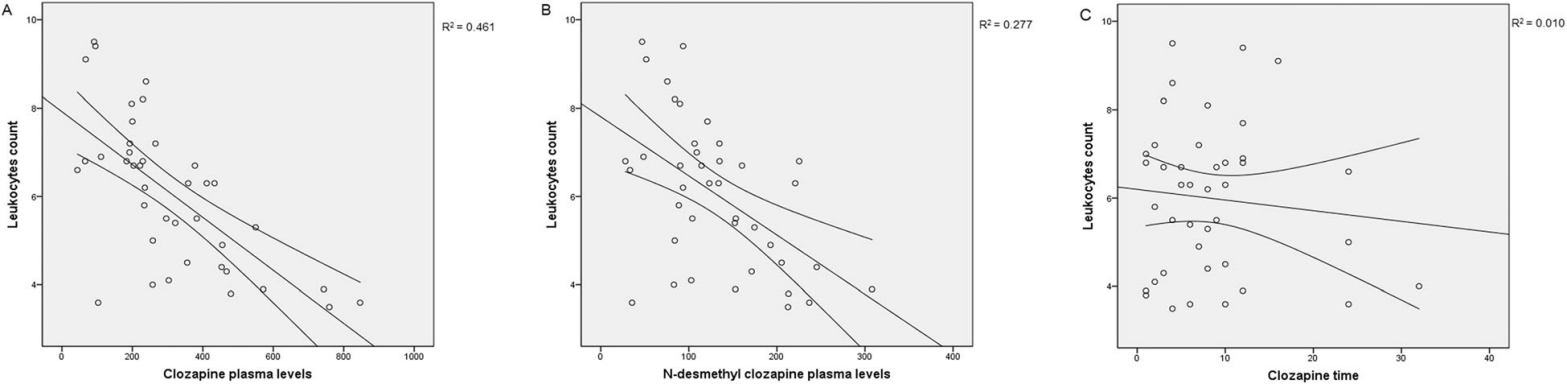
Scatter plot showing main relationships between Leukocytes counting and CLZ and DMC. Spearman’s and Pearson’s correlation coefficient and linear regression analysis were applied

After applying bivariate analyses, the plasma concentrations of CLZ and DMC were negatively correlated with both neutrophils and leucocytes counting (see Figs. 2 and 3 and Table 3 for bivariate correlation coefficients estimates). Also, the time taking CLZ and the CLZ dose were significantly associated to neutrophils and leucocytes counting, after bivariate analysis.

**Table 3:** Results from Sperman’s and Pearson’s correlations between biomarkers of CLZ and DMC

		Neutrophils	Leukocytes
CLZ plasma levels	Correlation coefficient *(p)*	(−0.703) (− 0.669)	(− 0.712) (− 0.679)
	*P*	(0.001) (0.001)	(0.001) (0.001)
DMC plasma levels	Correlation coefficient *(p)*	(−0.448) (− 0.538)	(− 0.476) (− 0.526)
	*P*	(0.004) (0.001)	(0.002) (0.001)

Multiple linear regression models were applied to data, in order to obtain parameter estimations for the independent determinants of the countings, taking as dependent variable either neutrophils or leucocytes counting (Table 4).

**Table 4:** Results from multiple regression analyses using biomarkers of CLZ and DMC as determinants of cell counting

Variable	Neutrophils		Leukocytes	
	Standardized Coefficient	*P*	Standardized Coefficient	*P*
CLZ plasma levels	−0.631	0.004	− 0.725	0.001
CLZ dose	−0.262	0.043	− 0.198	0.125
CLZ time	−0.267	0.024	− 0.271	0.023
DMC plasma levels	0.031	0.875	0.094	0.634

CLZ plasma concentrations, the time taking CLZ and CLZ dose remained significant determinants of neutrophils’ counting with a R^2^ = 0.447, using the multiple regression analysis model of Table 4. Likewise, the correlation of leucocyte counting vs CLZ plasma levels and CLZ time, showed a R^2^ = 0.461. As can be observed, DMC concentration was no longer an independent determinant of the countings (Table 4). In the case of leucocytes counting, time taking CLZ time is still significant after applying regression analysis, but CLZ dose was no longer a determinant.

A reduced number of patients in the study population were smokers, compared to other studies that support that more than 50% of patients with schizophrenia are smokers [16]. When tobacco smoking was included in the multiple linear model as a covariate, no relationship between neutrophils count, tobacco consumption, CLZ and DMC plasma levels were found, *p* = 0.893 and *p* = 0.905, respectively.

## Discussion

The method developed and validated here for CLZ and DMC plasma quantification showed to be selective, precise, with excellent recovery percentage. We showed that the method was well-suited for determining the drug and the metabolite, not only for the purpose of the present study, but to be used in clinical pharmacovigilance of this drug or to use it for other mechanistic studies.

The CLZ plasma levels observed in the sample of patients are in the rank positively associated with CLZ efficacy and below those related to overt toxicity observed in other studies [17, 18]. In this regard, we consider that the main result of the present study is the significant correlation observed between CLZ plasma levels and the neutrophils and leucocytes counting of the patients. In our opinion, this relationship is important, because the mechanism of clozapine-induced agranulocytosis, although unclear, has been suggested to be the result of the metabolism of the drug. In this regard, the metabolic transformation of CLZ to produce reactive oxygen species and nitrenium ion, is performed by NADPH oxidase/myeloperoxidase system, and CYP3A4, CYP2D6 and CYP1A2, mainly [19] and this biotransformation might be responsible for the decrease in the leukocytes count [20]. Other possibility is that CLZ itself may act at leucocytes to promote cells’ death by apoptosis, as reported by Bergemann et al. [21]. They found an 8-times increase of CLZ intracellular leucocytes concentration in patients suffering leukocytopenia, as compared to control patients without the adverse effect. Thus, CLZ may directly activate the nitrenium ion through two isoenzymes, then promoting hemotoxicity. The results presented here are in disagreement with those of Smith, et al. [22]. They found a positive correlation between neutrophils count and plasma DMC concentrations. Other authors have found no correlation between plasma CLZ and cells‘counting [23, 24]. Two factors may explain the differences between the results presented here and previous studies exploring the subject: 1) Methodological differences, as some studies are case reports of patients with agranulocytosis or longitudinal short-term studies 2) Obvious differences in populations’ genetics and a higher percentage of smokers in the study by Smith et al. may account for the different results obtained. As that study was retrospective, they recorded no data on the time the patients were taking the medication, a significant covariate in our study. They also employed two different methods to analyse plasma CLZ and DMC, making difficult to assess the reliability of the results. In contrast, our data were obtained after a careful validation of the analytical method employed. In the case of studies showing no correlation between plasma CLZ. In the case of.

It is important to remark that the dose of CLZ taken by patients was not a strong determinant of leucocyte counting in our study, as was plasma CLZ. This suggests that metabolism of CLZ, due to differences in CYP isoforms, may influence the effect of CLZ on neutrophils and leucocytes toxicity [25]. As a result of that, monitoring plasma CLZ itself may help in detecting a CLZ overdose with consequent decrease of neutrophil count. Drug monitoring of CLZ may also help psychiatrists to decision-making on CZP drug optimization and compliance.

The intensive pharmacovigilance must aim to monitor adverse reactions at any level, since a fatal adverse reaction can occur, such as the QT prolongation observed in this study. In this case, the patient’s levels of CLZ and DMC were, 152.90 ng/ml and 93.54 ng/ml, respectively, values above the average values of the study sample.

## Conclusion

In this study, we validated an analytical method by high performance liquid chromatography to measure plasma levels of CLZ, which allowed us to correlate plasma CLZ levels with the number of neutrophils and leukocytes. Likewise, the results showed that there is a negative correlation between the plasma levels of CLZ and the counting of blood neutrophils and leukocytes but not with DMC, suggesting that neutrophils and leukocytes toxicity is associated to CLZ metabolism. As the dose of CLZ taken by the patient was a poor determinant of leucocyte counting, it is of great importance to adjust CLZ doses by using CLZ monitoring, in order to maintain safe therapeutic dosing without the presence of adverse effects.

## Data Availability

The datasets used and/or analyzed during the current study are available from the corresponding author on reasonable request.

## Abbreviations

CLZ: Clozapine
DMC: N-desmethyl-clozapine
FGA: First-generation agents
SCID: Structured Clinical Interview
SGA: Second-generation antipsychotics
